# Quantitative accuracy of ^177^Lu SPECT reconstruction using different compensation methods: phantom and patient studies

**DOI:** 10.1186/s13550-016-0172-0

**Published:** 2016-02-18

**Authors:** Eero Hippeläinen, Mikko Tenhunen, Hanna Mäenpää, Antti Sohlberg

**Affiliations:** HUS Medical Imaging Center, Helsinki University Central Hospital, POB 340, FI-00029 HUS Helsinki, Finland; Department of Physics, University of Helsinki, P.O. Box 64, FI-00014 Helsinki, Finland; Department of Oncology, Cancer Center, Helsinki University Hospital, POB 180, FI-00029 HUS Helsinki, Finland; Department of Nuclear Medicine, Joint Authority for Päijät-Häme Social and Health Care, Keskussairaalankatu 7, FI-15850 Lahti, Finland; HERMES Medical Solutions, Stockholm, Sweden

**Keywords:** Image reconstruction, Quantitation, ^177^Lu, PRRT, OSEM, Time-activity curve

## Abstract

**Background:**

In targeted radionuclide therapy (TRT), accurate quantification using SPECT/CT images is important for optimizing radiation dose delivered to both the tumour and healthy tissue. Quantitative SPECT images are regularly reconstructed using the ordered subset expectation maximization (OSEM) algorithm with various compensation methods such as attenuation (A), scatter (S) and detector and collimator response (R). In this study, different combinations of the compensation methods are applied during OSEM reconstruction and the effect on the ^177^Lu quantification accuracy is studied in an anthropomorphic torso phantom. In addition, the phantom results are reflected to (177)Lu-DOTA-Tyr3-octreotate (^177^Lu-DOTATATE)-treated patient data and kidney absorbed dose estimates.

**Methods:**

The torso phantom was imaged with nine various sized (0.4–104.4 cm^3^) spherical inserts, filled with known ^177^Lu activity ranging from 0.5 to 105.5 MBq. Images were reconstructed using OSEM algorithm using A, AR and ARS compensation method combinations. The compensation method combinations were compared by calculating the concentration recovery coefficient (cRC) for each insert. In addition, ten ^177^Lu-DOTATATE-treated patient’s post-therapy dosimetry acquisitions were reconstructed, and the absorbed dose to kidneys was estimated.

**Results:**

cRC values depend on the insert size for all compensation methods. AR and ARS produced significantly higher cRC values than attenuation correction alone. There were no cRC value differences between the methods for the smallest 1-cm-diameter insert, cRC being 0.18. However, the collimator and detector response compensation method (R) made the 1.3-cm-diameter insert clearly visible and improved cRC estimate from 0.19 to 0.43. ARS produced slightly higher cRC values for small- and medium-sized inserts than AR. On the patient data, a similar trend could be seen. AR and ARS produced higher kidney activities than using attenuation correction alone; the total absorbed doses to the right and left kidneys were on average 15 and 20 % higher for AR and 19 and 25 % higher for ARS, respectively. The effective half-life decay estimated from time-activity curves however showed no notable difference between the compensation methods.

**Conclusions:**

The highest cRC values were achieved by applying ARS compensation during reconstruction. The results were notably higher than those using attenuation correction alone. Similarly, higher activity estimates and thus higher absorbed dose estimates were found in patient data when all compensation methods were applied. ARS improved cRC especially in small-sized sources, and it thus might aid tumour dosimetry for ^177^Lu PRRT treatments.

## Background

The ^177^Lu-labelled somatostatin analogues (177)Lu-DOTA-Tyr3-octreotide (^177^Lu-DOTATOC) and (177)Lu-DOTA-Tyr3-octreotate (^177^Lu-DOTATATE) are used for peptide receptor radionuclide therapy (PRRT) for the treatment of neuroendocrine tumours (NETs), which are a group of tumours characterized by overexpression of somatostatin receptors. Although both radiopharmaceuticals have been reported to have very high affinity to somatostatin receptors [1] and high tumour accumulation [2], kidney doses have also been reported to be high and widely variable between patients [2, 3]. Therefore, patient specific post-therapeutic dosimetry should be performed to ensure patient safety. Dosimetry should be extended from the kidney alone to the tumour as demonstrated in Ilan et al. [4] to further our understanding of the PRRT dose response as well as to target the treatments to specific patients who could benefit from them.

Absorbed dose calculation of ^177^Lu-labelled radiopharmaceuticals is based on activity distribution quantification using single photon emission imaging combined with computed tomography (SPECT/CT). In addition to highly ionizing beta radiation that produces about 96 % of the absorbed dose [5], ^177^Lu also emits low and medium energy *γ* radiations that can be imaged using a SPECT/CT system. Activity quantification from SPECT images is not a trivial task. Inaccuracies in image reconstruction and thus in radionuclide uptake estimates are introduced through photon attenuation, scattering, and collimator blurring. Iterative reconstruction techniques allow effective methods for compensation, namely attenuation correction (A), scatter correction (S) and collimator-detector response (R) compensation [6–8], and thus are used in different combinations in quantitative targeted radionuclide therapy (TRT) imaging.

The ordered subsets expectation maximization (OSEM) algorithm [9] has been widely used for quantitative reconstruction and internal dosimetry calculations. It has been studied using different combinations of compensation methods, and average quantification error has been reported to be from low (1 %) to moderate (20 %) [10]. The results depend on many factors such as source size and shape, isotope and activity concentration of source and background, which increases complexity and also makes comparison between published results difficult. ^177^Lu radionuclide quantification accuracy has not been studied very extensively [11–14] even though a large number of patients have been treated with ^177^Lu-labelled somatostatin analogues. Recently, Sanders et.al [12] validated a ^177^Lu quantitative SPECT/CT imaging protocol in vivo using urine samples, implementing the method published earlier by Zeintl et al. [15]. In the work of Sanders et.al, the mean error in activity concentration estimated from patients using SPECT/CT was 10.1 ± 8.3 % (range, −19.4 to 22.4 %). The activity estimates were done at one time point after ^177^Lu-DOTATATE therapy injection, and no estimation was made of the effect of degraded image statistics at later time points on the quantification accuracy. Sanders et.al concluded that quantitative SPECT/CT in vivo is feasible but could benefit from improved reconstruction methods and a more sophisticated scatter compensation method [12].

In this study, OSEM-based reconstruction package was validated for ^177^Lu radionuclide using realistic anthropomorphic torso phantom measurements. The quantitation accuracy of the reconstruction package was determined using different sized spherical inserts in the torso phantom. Concentration recovery coefficients (cRCs) were calculated for the inserts through application of state of the art compensation methods such as Monte Carlo-based scatter correction (S), collimator-detector response (R) and CT-based attenuation correction (A) during the reconstruction. We also studied how different compensation combinations affect the fundamental dosimetric parameters such as the effective half-life and absorbed dose estimates using clinical ^177^Lu-DOTATATE-treated patient data.

## Methods

Our study is composed of two different parts: (i) determining the ^177^Lu quantification accuracy of our reconstruction package using a torso phantom and (ii) reconstruction of patient data with different compensation combinations and determination of absorbed dose estimates for kidneys. All the phantom and patient data were acquired, reconstructed and analysed in a similar way, and the details are given in the following sections.

### SPECT/CT imaging protocol

Images were acquired on a SPECT/CT system (Symbia T2, Siemens Healthcare, Erlangen, Germany) using the local clinical imaging protocol for ^177^Lu-therapy patients. The SPECT acquisition included the following parameters: 64 projections, 20 s per projection, a non-circular step-and-shoot acquisition orbit, 128 × 128 matrix and 4.8 mm pixel size. Projection data were acquired using a 20 % energy window centred on the 208 keV photopeak using a parallel-hole medium energy low penetration (MELP) collimator. After the SPECT acquisition, a CT scan was performed using the CT tube voltage of 130 keV and a quality reference tube current-time product of 100 mAs (CareDose 4D). The calculated CTDI_vol_ was 10.8 mGy, and the CT scans covered one or two SPECT field of views (40 cm per view) depending on the case. CT data was reconstructed to 512 × 512 matrix with 0.98 × 0.98 mm pixel size and 3.0 mm slice thickness. The CT reconstruction was performed using two different kernels: smoother kernel (B08s) for attenuation correction and scatter correction and sharper kernel (B35) for volume of interest (VOI) delineation.

### Image reconstruction

All images were reconstructed with an OSEM algorithm with 15 iterations and 16 subsets. Compensations for attenuation and collimator-detector response were applied as described in [16]. Briefly, the CT attenuation map was generated using a bilinear conversion for HU to linear attenuation coefficient values, and collimator correction was based on the Gaussian convolution. The scatter correction was implemented using an accelerated Monte Carlo (MC) simulation method [17]. Because MC scatter simulations are time-consuming, the scatter correction was accelerated with convolution-based forced detection and calculation of the scattering into a sparser matrix than the resulting image. The scatter estimate was calculated for only the first two iterations, and the scattering was modelled only during forward projection.

All SPECT reconstructions produced images with 128 × 128 matrix with 128 slices and 4.8 × 4.8 × 4.8 mm^3^ voxel size. Each data set was compensated separately for attenuation (A), attenuation and collimator-detector response (AR) and attenuation, collimator-detector and scatter (ARS). Attenuation and scatter correction (AS) was not implemented, because the convolution-based forced detection scatter correction method inherently includes collimator and detector modelling. We think that omitting collimator-detector modelling for primary photons but applying it for scatter photons is illogical. Post-filtering was not applied to any of the data presented in this study. The reconstruction methods used in this work were implemented into the reconstruction engine of a commercially available reconstruction package, HERMES HybridRecon version 1.1 (HERMES Medical Solutions AB, Stockholm, Sweden).

### Phantoms

#### Torso phantom

**Fig. 1:**
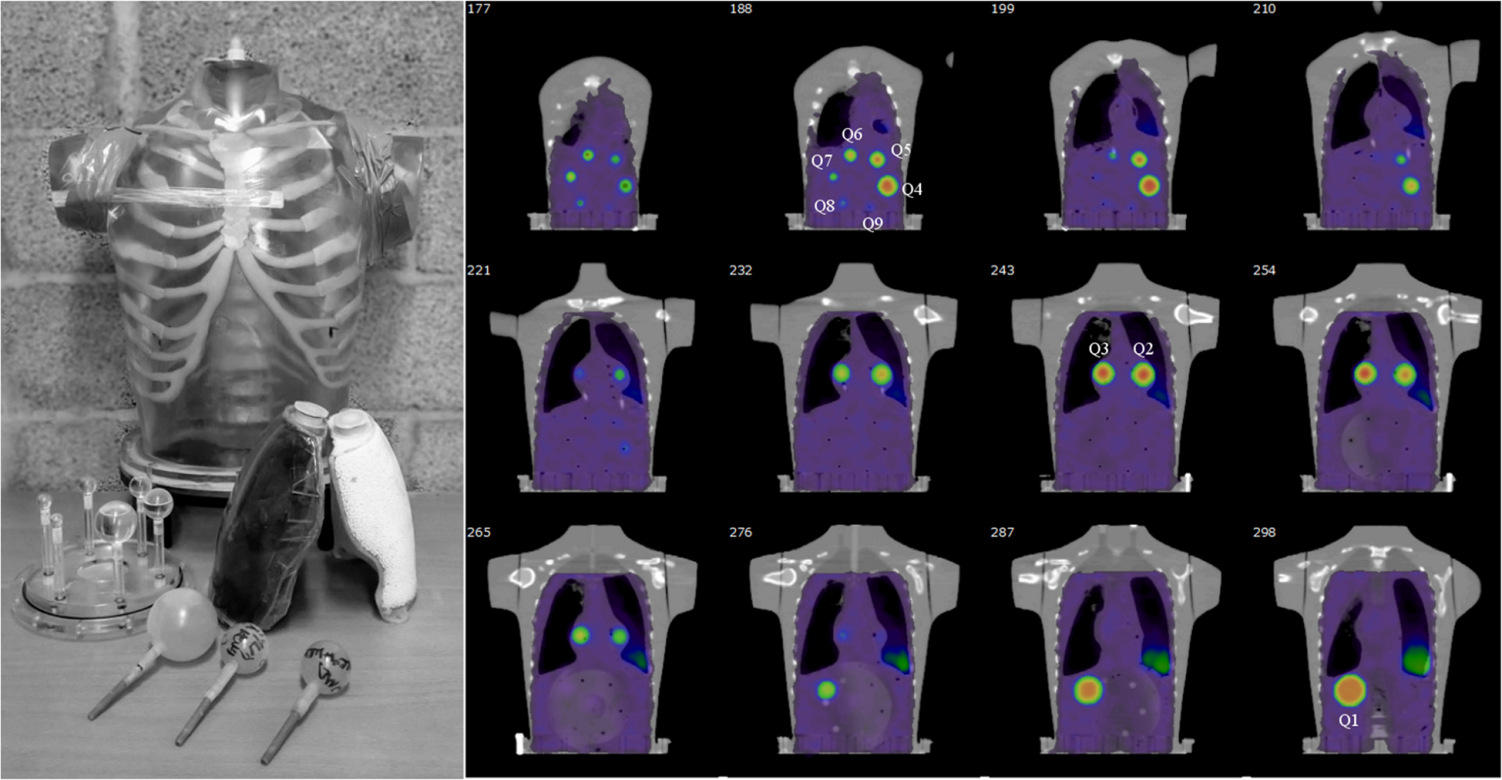
A torso phantom (*left*) and nine spherical inserts (*right*). Seven of the inserts (Q1, Q4–Q9) are located in the abdomen. Two of the inserts (Q2 and Q3) were located next to the lungs under the sternum

A RSD Alderson phantom (torso phantom) was modified to include nine spherical inserts modelling tumours and two lung density structures, in order to evaluate the accuracy of the reconstruction methods for inhomogeneous density conditions. Six of the inserts were part of the NEMA image quality phantom, and three were produced in-house. The inserts were filled with ^177^Lu solution and placed into the torso phantom. Two identical inserts (Q2 and Q3) were placed next to the lungs, and others were placed in the abdomen (Table 1). The right non-perfusable lung had density of 0.3 g/cm^3^. The left lung was an open-cell foam type with low ^177^Lu activity filling with a density of 0.3 g/cm^3^. The volumes of the right and left lung shells were 1134 and 907 cm^3^, respectively (Fig. 1).

**Table 1:** The volume and activities as well as the locations of the tumour inserts in the torso phantom

Label	Volume (cm^3^)	Activity (MBq)	Location in phantom
Q1	104.4	105.2	Abdomen
Q2	30.3	31.2	Next to active left lung
Q3	30.3	30.4	Next to non-active right lung
Q4	26.1	27.2	Abdomen
Q5	10.8	11.9	Abdomen
Q6	5.5	5.8	Abdomen
Q7	2.6	2.7	Abdomen
Q8	1.2	1.2	Abdomen
Q9	0.4	0.5	Abdomen

After inserting lung and spherical inserts, the phantom was filled with ^177^Lu activity to make imaging and analysis processes more representative of patient studies. The inserts and left lung concentrations were 30 and 1.7 times greater than the background, respectively. The total activity in the phantom was 806 MBq during imaging.

#### Calibration phantom

The SPECT/CT scanner was calibrated using a calibration phantom as suggested in MIRD Pamphlet no. 23 [10]. A bottle (diameter 6.6 cm, height 15.3 cm and volume of 525 cm^3^) was filled with homogeneous ^177^Lu solution with an activity concentration of 0.45 MBq/cm^3^, and it was placed inside the cylindrical body of the Jaszczak phantom filled with water. For each reconstruction parameter set, a VOI was drawn manually over the bottle based on the dimensions of the bottle and the CT images. The volumetric sensitivity *Ŝ*_vol_ (kBq/ml) of the SPECT/CT system was determined as reported by Sanders et.al [12]. During the study, the *Ŝ*_vol_ values were used to convert torso phantom and patient SPECT images to activity concentration distributions.

### Patient data

SPECT/CT data of ten different patients were selected randomly and retrospectively from over 100 who underwent treatment with ^177^Lu-DOTATATE, at the Helsinki University Hospital, Cancer Center between 2011 and 2015. Patients were diagnosed with progressive metastatic neuroendocrine tumour (grades I–II). The ten selected patients were treated with infusion of ^177^Lu-DOTATATE (range of activity being from 3.4 to 8.1 GBq) incorporated with kidney protective amino acid infusion. For each three successive SPECT/CT studies were acquired at 24, 48 and 168 h after the infusion and were reconstructed for this project in order to study time-activity curves (TACs). The patient data is summarized in Table 2.

**Table 2:** Patient data characteristics

Subject	Activity (GBq)	SPECT/CT imaging time (h)			Delineated kidney VOI volumes (ml)	
		1.	2.	3.	Left	Right
P1	7.5	25	52	173	140	132
P2	8.1	25	120	168	281	285
P3	7.2	24	47	168	165	149
P4	7.3	24	45	168	287	270
P5	6.0	33	110	158	137	131
P6	3.7	38	133	181	182	178
P7	8.0	27	117	164	193	154
P8	8.0	28	69	164	176	148
P9	8.1	23	120	168	115	127
P10	4.1	24	71	146	162	134

### Data analysis and absorbed dose calculations

Nine spherical VOIs were delineated based on the insert volumes and physical boundaries seen in the CT images. Sizes of the delineated volumes are shown in the Table 1. The activity concentration measured using the VOIs was evaluated using a concentration recovery coefficient (cRC), which was defined as:

(1) $$ cRC=\frac{\left(\frac{R_{\mathrm{insert}}}{V_{\mathrm{insert}\ }{\widehat{S}}_{\mathrm{vol}}}\right)}{C_{\mathrm{insert}}}, $$

where *R*_insert_ is the count density within the defined VOI, V_insert_ is the VOI volume and C_insert_ is the true concentration in the insert. cRC was calculated for each insert and each compensation method.

**Fig. 2:**
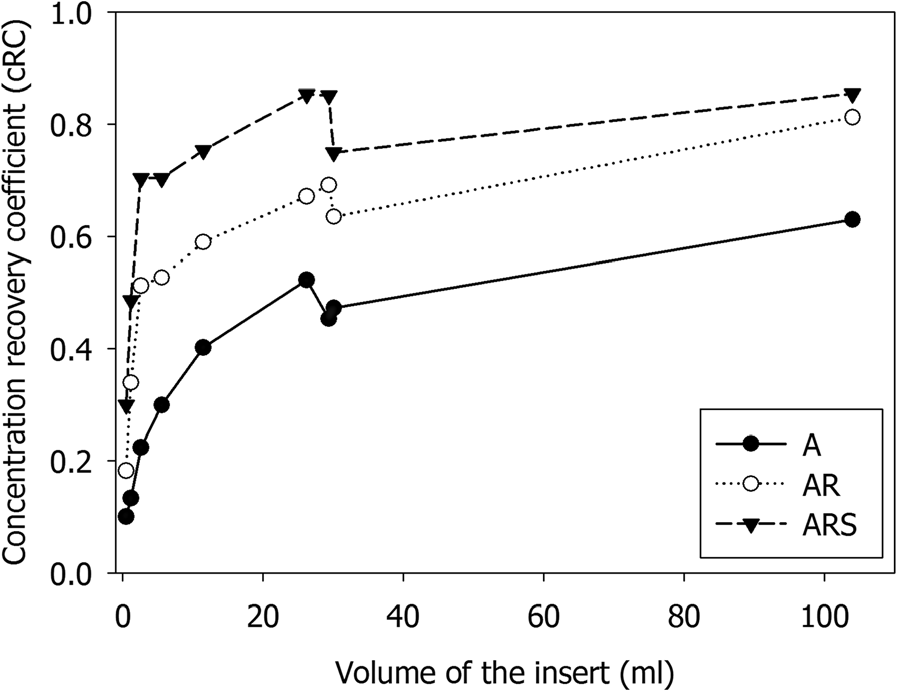
cRC for A, AR and ARS methods as a function of spherical insert volume

The data for each patient was reconstructed the same way as the phantoms (15 iterations, 16 subsets) using A, AR and ARS compensation methods. All the reconstructed images were co-registered to the 24-h ARS-reconstructed SPECT image using mutual information-based co-registration algorithm. The registrations were each visually verified. CT-based VOIs were drawn using the physical boundaries of each kidney, excluding the renal pelvis (Fig. 2). TACs were produced based on image statistics. A mono-exponential function *y*(*t*) = *A*_0_*e*^− *λt*^) was fitted to the TACs, and the effective half-life ${T}_{\mathrm{eff}}=\frac{ \ln 2}{\lambda }$ was calculated using the decay constant *λ*, obtained from the exponential fit.

The total number of decays within the kidney volume was determined as an analytical integral of the fitted exponential function from the time of injection (*t* = 0) to infinity. The total number of decays was converted to absorbed dose by taking into account only the self-dose produced by electrons [5]. The absorbed dose estimates, *D*, were compared between compensation method combinations.

## Results

### The torso phantom study

The cRC results for A, AR and ARS compensation combinations as a function of the insert volume are shown in Fig. 2. From this figure, we can see how cRC is dependent on the size of the insert because of the limited spatial resolution of the SPECT system. Since the collimator-detector response compensation method can partly compensate for the resolution loss the dependence gets weaker as more compensation methods are applied during reconstruction. The ARS method produced the highest recovery values for all spheres; the gain was dependent on the insert size and ranged from 200 % for small inserts to 35 % for the largest inserts when compared to A method. None of the methods could recover the true concentration (cRC = 1) for any of the inserts, because VOIs were drawn based on the CT image and physical boundaries and no partial volume correction was attempted.

### Patient data

**Fig. 3:**
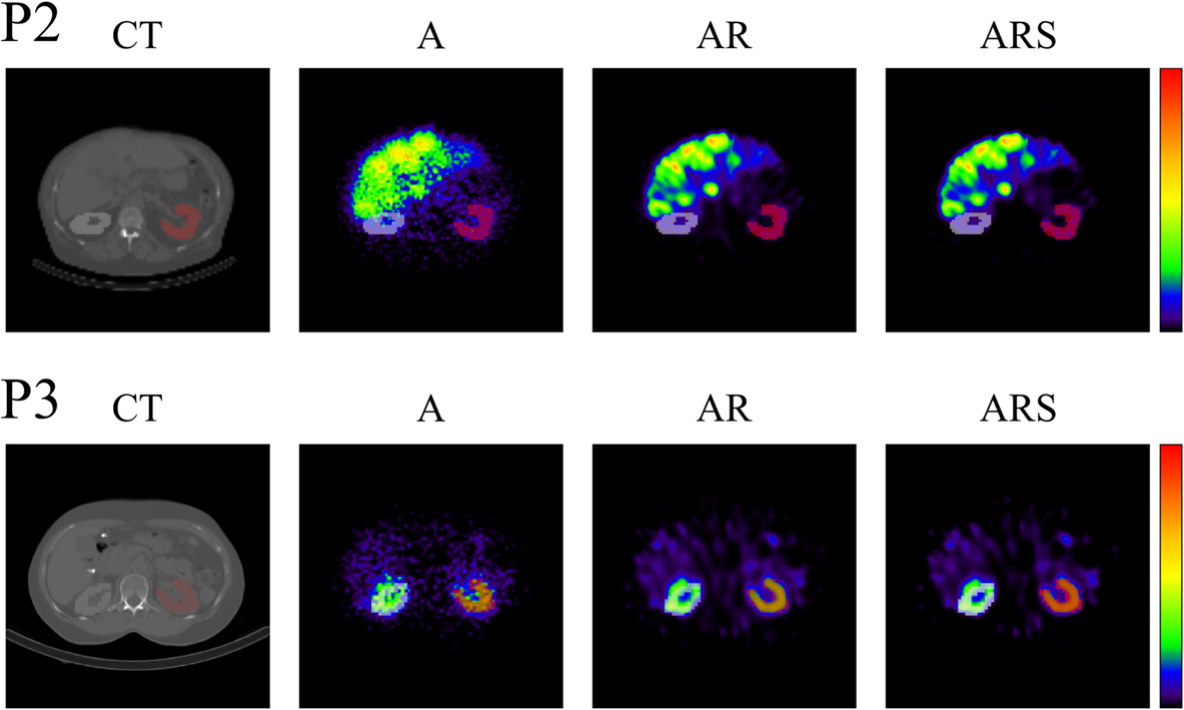
Representative transverse SPECT/CT slices reconstructed using A, AR and ARS compensation methods of P2 and P3 subjects treated with ^177^Lu-DOTATATE

Representative transverse SPECT/CT slices reconstructed using A, AR and ARS compensation methods are shown in Fig. 3. The samples are from patients 2 and 3 who had markedly different amounts of activity accumulation in the liver as a result of metastases in patient 2. Slices reconstructed with A were quite noisy, because no post-filtering was applied after reconstruction. The use of post-reconstruction filtering would have made these images visually more appealing but would have also blurred images and reduced spatial resolution. Slices produced with AR and ARS are significantly smoother than the A images due to the collimator-detector response compensation method.

**Fig. 4:**
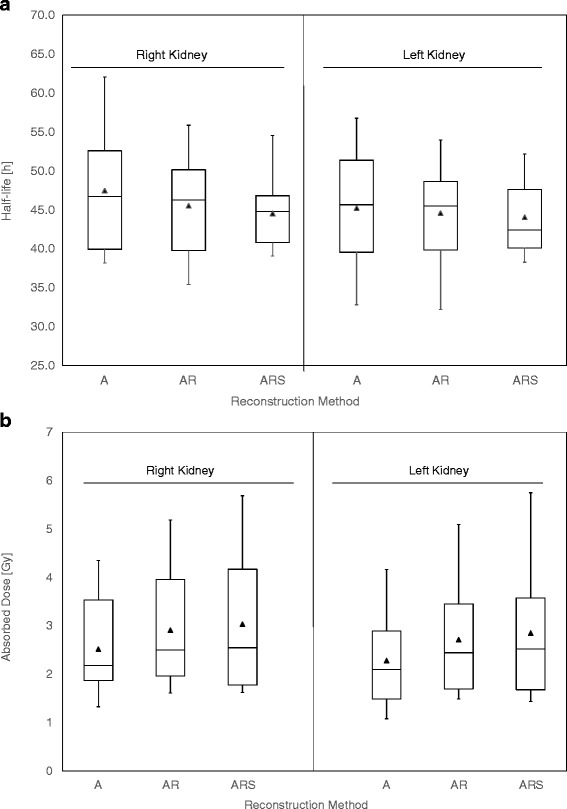
Box plots of effective half-live (*above*) and absorbed dose estimates (*below*) to the right and left kidneys for ten different patient cases. The *full triangles* indicate average values of the set and the *whiskers* are extended to minimum and maximum data values (Spear style)

The effective half-lives and absorbed dose estimates are shown in Fig. 4. There was no clear trend on how effective half-life depended on the different compensation methods. On average, AR and ARS produced 3 and 4 % lower effective half-lives than A. On the other hand, AR method produced on average 15 and 20 % and ARS 19 and 25 % higher higher absorbed doses to the right and left kidneys than A due to systematically higher activity estimates at different acquisition time points. There was only one patient case (P2 right kidney) where AR and ARS methods produced smaller absorbed dose estimates than method A. This can be explained partly by the better resolution due to collimator compensation method and partly by scatter correction which both helped to reduce spill over from the highly metastatic liver to the right kidney (Fig. 3).

## Discussion

In this study, we have validated the quantitative accuracy of our reconstruction package for ^177^Lu using an anthropomorphic phantom. We determined cRC values from nine spherical sources with varying sizes using different compensation method combinations applied during reconstructions. Applying collimator-detector response compensation dramatically improves cRC compared with the attenuation corrected only images. In addition to the collimator-detector response compensation method, the MC simulation-based scatter correction method improved quantitation accuracy even more, especially for small sources.

Our results are comparable with recently published studies [12, 14]. In a recent study, Sanders et al. reported the cRC being around 80 % for the 16-ml spherical source, while we found cRC to be 75 and 85 % for sources with size of 11.5 and 26.2 ml, respectively (Fig. 2). For the small inserts like Q7 with volume of 2.6 ml, the ARS method produced cRC of 70 %, which is as high as Sanders et.al reported for the sources with volume from 4 to 8 ml.

From the patient data, we found a similar trend in absorbed doses as with the cRC values: ARS produced the highest absorbed dose estimates between the methods evaluated in this study due to systematically higher activity estimates at every acquisition time point. For example in eight out of ten cases, ARS produced over 20 % higher estimated absorbed doses into the left or right kidney than method A, and in patient P9 case, ARS produced up to 40 % higher estimated dose than method A. In general, the absorbed doses to kidneys ranged from 1.3 to 5 Gy. Similar dose ranges have been reported earlier [2, 3]. Considering 23 Gy absorbed dose limit to the kidneys derived from external radiotherapy, the difference between reconstruction methods can be considered to be clinically relevant. In other words, for an individual patient, the number of treatment cycles can depend on what reconstruction method has been used during dose calculations.

Frequently, in patients with liver metastatic neuroendocrine tumours, the distinction between the right kidney and liver metastases’ ^177^Lu-DOTATATE activity distributions is challenging to make due to the poor spatial resolution of SPECT systems. In this study, we have shown how the collimator-detector compensation method can help with these kinds of cases. In patient P2, ARS produced 24 % smaller absorbed doses to the right kidney than A, but it was clearly due to an improved distinction between kidney and liver activity distributions and reduced spill in from liver volume to the kidney.

In our study, we have delineated the VOIs based on CT image and tracked the physical boundaries of either inserts or kidneys. The method is arguably cumbersome and sensitive to partial volume effect (PVE). Some of the authors of previous studies have used a so-called a small VOI method [7], where a small VOI is used to sample activity within the whole kidney. The small VOI method is easy to implement and is at least justifiable for cases like the patient P2, where a clear distinction between activity distributions is difficult to make. However, the small VOI gives a limited sample of the kidney’s activity distribution, and thus, the dose calculation is carried out using a small and manually selected representative VOI statistics. Because the selection of the small VOI is not always unambiguous, in clinical practice, dose calculation is compromised, for example, to represent only the maximum dose of the kidney, and it does not take activity heterogeneity into account. In addition to this, to carry out the patient-specific dose calculation, the kidney masses should be delineated patient specifically; thus, whole kidney delineation was preferred in our study.

## Conclusions

Applying attenuation, collimator-detector and Monte Carlo scatter compensation methods (ARS) improved cRC especially in small-sized sources which might aid tumour dosimetry for ^177^Lu PRRT treatments. From the patient data, we observed that ^177^Lu-DOTATATE half-life estimates did not apparently depend on reconstruction compensation methods.
